# Bioleaching of Manganese Oxides at Different Oxidation States by Filamentous Fungus *Aspergillus niger*

**DOI:** 10.3390/jof7100808

**Published:** 2021-09-28

**Authors:** Bence Farkas, Marek Bujdoš, Filip Polák, Michaela Matulová, Martin Cesnek, Eva Duborská, Ondřej Zvěřina, Hyunjung Kim, Martin Danko, Zuzana Kisová, Peter Matúš, Martin Urík

**Affiliations:** 1 Institute of Laboratory Research on Geomaterials, Faculty of Natural Sciences, Comenius University in Bratislava, Mlynská Dolina, Ilkovičova 6, 842 15 Bratislava, Slovakia; farkas62@uniba.sk (B.F.); marek.bujdos@uniba.sk (M.B.); michaela.matulova@uniba.sk (M.M.); eva.duborska@uniba.sk (E.D.); peter.matus@uniba.sk (P.M.); 2 Department of Soil Science and Soil Protection, Czech University of Life Sciences Prague, Kamýcká 129, 16500 Praha-Suchdol, Czech Republic; polakf@af.czu.cz; 3 Department of Nuclear Reactors, Faculty of Nuclear Sciences and Physical Engineering, Czech Technical University in Prague, V Holešovičkách 2, 18000 Prague, Czech Republic; martin.cesnek@fjfi.cvut.cz; 4 Department of Public Health, Faculty of Medicine, Masaryk University, Kamenice 753/5, 62500 Brno, Czech Republic; zverina@med.muni.cz; 5 Department of Mineral Resources and Energy Engineering, Jeonbuk National University, Jeonju-si 54896, Korea; kshjkim@jbnu.ac.kr; 6 Department of Environment and Energy, Jeonbuk National University, Jeonju-si 54896, Korea; 7 Polymer Institute, Slovak Academy of Sciences, Dúbravská Cesta 9, 845 41 Bratislava, Slovakia; upoldan@savba.sk; 8 Institute of Molecular Biology, Slovak Academy of Sciences, Dúbravská Cesta 21, 845 51 Bratislava, Slovakia; zuzana.kisova@savba.sk

**Keywords:** bioextraction, bioleaching, filamentous fungi, manganese oxide, oxalate

## Abstract

This work aimed to examine the bioleaching of manganese oxides at various oxidation states (MnO, MnO·Mn_2_O_3_, Mn_2_O_3_ and MnO_2_) by a strain of the filamentous fungus *Aspergillus niger*, a frequent soil representative. Our results showed that the fungus effectively disintegrated the crystal structure of selected mineral manganese phases. Thereby, during a 31-day static incubation of oxides in the presence of fungus, manganese was bioextracted into the culture medium and, in some cases, transformed into a new biogenic mineral. The latter resulted from the precipitation of extracted manganese with biogenic oxalate. The Mn(II,III)-oxide was the most susceptible to fungal biodeterioration, and up to 26% of the manganese content in oxide was extracted by the fungus into the medium. The detected variabilities in biogenic oxalate and gluconate accumulation in the medium are also discussed regarding the fungal sensitivity to manganese. These suggest an alternative pathway of manganese oxides’ biodeterioration via a reductive dissolution. There, the oxalate metabolites are consumed as the reductive agents. Our results highlight the significance of fungal activity in manganese mobilization and transformation. The soil fungi should be considered an important geoactive agent that affects the stability of natural geochemical barriers.

## 1. Introduction

Manganese is a transition metal that is the 10th most abundant chemical element on Earth and makes up to 0.1% of the Earth’s crust. It is a structural component of more than 250 minerals and, in the case of several other minerals, is a substituent of Fe^2+^ and Mg^2+^ cations [1].

Manganese occurs in various oxidation states of which the +II, +III and +IV are predominant under natural conditions [2]. In the soil environment, it occurs in dissolved form, and it is partially adsorbed onto the surface of non-manganese mineral phases or organic compounds, or it is immobilized in organisms. Most of the manganese in the soil is found as a component of primary mineral phases or is bound to secondary minerals [3]. Baranowsky et al. [4] showed that soil manganese is primarily associated with fractions of carbonates and metal oxides. However, significant amounts can also be found in residues left after sequential extraction. A similar distribution has been found in sediments, where the manganese was reported to be bound mostly to carbonates and hydrated oxides [5,6].

Manganese oxides and (oxo)hydroxides are among the most common secondary minerals of manganese in the soil. These are reactive phases that play an important role in the geochemical cycling of various elements. They generally participate in redox transformations of organic and inorganic compounds, since they represent the strongest oxidizing agents found in the environment [7].

As an essential nutrient, manganese is involved in various cellular functions such as redox homeostasis, metabolism of sugars and protein synthesis; and it is incorporated in vital molecules such as vitamin B or Mn-superoxide dismutase [8]. However, it becomes hazardous upon exposure to high concentrations with severe adverse impacts on plants and soil microorganisms [9]. Therefore, upon exposure to elevated concentrations, microorganisms are capable of utilizing various strategies to immobilize manganese to limit its bioavailability [10].

The biosynthesis of manganese oxides in soils and sediments contributes to the formation of geochemical barriers, the high sorption and redox capacity of which can significantly decrease the mobility of potentially toxic elements in the environment [11]. Since the stability of manganese oxides is essential for limiting the spread of contamination, the microbially induced biodeterioration of these phases [2] should be studied in great detail, and carefully monitored in soils, especially in regard to the activity of various geoactive agents of biological origin [12,13,14]. First, however, controlled laboratory-scale experiments are required for a better understanding of the processes leading to the manganese oxides’ deterioration.

Our research is therefore based on the assumption that the MnO, MnO·Mn_2_O_3_, Mn_2_O_3_ and MnO_2_, which are constituents of naturally occurring geochemical barriers, should react differently with the metabolites of *Aspergillus niger*, a common soil filamentous fungus. We evaluated the contribution of the fungus to the chemical biodeterioration of manganese oxides using advanced analytical and mineralogical techniques. They allowed us to observe the kinetics of this fundamental process and led us to a better understanding of mechanisms that microorganisms employ to affect the manganese geochemistry in the environment.

## 2. Materials and Methods

### 2.1. Fungal Strain

The fungus *Aspergillus niger* strain CBS 140837 was obtained from the fungal collection of the Department of Mycology and Physiology at the Institute of Botany, Slovak Academy of Sciences. The fungal strain was maintained on the Sabouraud agar plates at 25 °C.

### 2.2. Manganese Toxicity

The manganese 50% inhibitory concentration for the fungal radial growth was examined using various concentrations of manganese(II) (100; 250; 450; 650 and 1000 mg·L^−1^), prepared by dissolving MnCl_2_·4H_2_O (Centralchem, Bratislava, Slovakia) in the Sabouraud Dextrose Agar (HiMedia, Mumbai, India). The media were inoculated with *A. niger* strain and were cultivated at 25 °C for 7 days. We measured the radial growth of the fungus daily, and it was used to calculate the fungal growth parameters using the modified Gompertz growth equation [15]. Then, the 50% inhibitory concentration for the selected growth parameter was estimated using probit analysis.

### 2.3. Bioextraction of Manganese

The 31-day long manganese extraction experiments using the *A. niger* strain were performed in 100 mL sterile Erlenmeyer flasks with the mixture of 50 mL Sabouraud Dextrose Broth culture medium (HiMedia, Mumbai, India) and 0.635 g of MnO, 0.600 g of MnO·Mn_2_O_3_, 0.826 g of Mn_2_O_3_, or 0.844 g of MnO_2_. However, the initial extraction tests were performed with the 0.250 g weight of each manganese oxide for a period of 21 days. The manganese oxides in various oxidation states (MnO, MnO_2_, Mn_2_O_3_ and MnO·Mn_2_O_3_) were obtained from Sigma-Aldrich (Darmstadt, Germany).

To inoculate the growth media, a spore suspension was produced by washing the surface of a 10-day-old *A. niger* culture with sterile water, and approximately 10^5^ CFU were used as inoculum. Prior to inoculation, the manganese oxides in 100 mL Erlenmeyer flasks were sterilized by dry heating and, after sterilization, the culture medium was added. The control experiments were performed using a manganese oxide-free cultivation of fungus or non-inoculated control.

All experiments were carried out under laboratory conditions for the 31-day long static cultivation that allowed fungus to reach the stationary growth phase. During the cultivation, the pH of the culture medium, as well as the dissolved manganese and organic acid content, were determined 21 times over the cultivation period. The spent medium was filtered, and the pH was recorded. The harvested biomass was collected, washed with distilled water, sonicated and mechanically treated to remove residual manganese oxalate crystals, dried in a laboratory oven at 70 °C and weighed. Furthermore, the structural and morphological properties of mineral phases collected at the end of cultivation were characterized. In the case of the initial 21-day long tests, only the total extracted manganese was measured in the culture medium.

### 2.4. Analytical Procedures

The culture media filtrates were analyzed for the total manganese content by flame atomic absorption spectroscopy (F AAS) at line Mn 279.5 nm using an AAS spectrometer Perkin-Elmer Model 1100 (Perkin-Elmer, Uberlingen, Germany). Deuterium background correction was used. Calibration solutions were prepared from CertiPUR ICP 1000 mg·L^−1^ single-element standard solutions (Merck, Darmstadt, Germany). To confirm the method’s accuracy, the CRM Astasol AN9090MN (aqueous multi-element standard solution, Analytika, Praha, Czech Republic) was used. The CRM’s quantification was within the certified ranges. The precision of the method was 4% for Mn (relative combined standard uncertainty with coverage factor k  =  2).

The low-molecular-weight organic acids in the culture medium were analyzed by capillary isotachophoresis. Analysis of anions in 10 mL filtrate (0.45 µm) from the culture medium collected in the desired time intervals was performed by a ZKI 01 isotachophoretic analyzer (Villa Labeco, Spišská Nová Ves, Slovakia) using the itp-itp mode. The 10 mmol·L^−1^ hydrochloric acid adjusted with β-alanine to pH 4.25 plus 0.1% methylhydroxylethylcellulose was used as the leading electrolyte, and 5 mmol·L^−1^ caproic acid with histidine was used as the terminating electrolyte. For the quality control, a Quality Control Standard (QCS) was analyzed before each batch sample analysis. QCS (200 μM oxalate, citrate, gluconate, acetate) was prepared by dilution of 1000 mg·L^−1^ batch solutions of anions (TraceCERT Oxalate, Citrate, Gluconate, Acetate IC Standards; Sigma Aldrich, Saint-Louis, MO, USA). The isotachopherograms were evaluated by the analyzer supplied with ITPWin ver. 2.30 software [16] (Villa Labeco, Spišská Nová Ves, Slovakia).

Manganese oxides and their transformants were analyzed by high energy X-ray diffraction (HEXRD). The experiment was performed at beamline P02.1 of PETRA III electron storage ring (DESY, Hamburg, Germany). Energy of the beam was set to *E* = 59.73091 keV (*λ* = 0.20757 Å). The samples were put into Kapton tubes with a diameter of 1 mm and were illuminated by an incident beam that had a cross section of 0.5 mm× 0.5 mm. All the diffraction patterns were collected at room temperature using a two-dimensional detector Perkin Elmer 1621 (2048 × 2048 pixels, pixel size 200 μm × 200 μm). The sample-to-detector distance (SDD) was set to 1285 mm. A CeO_2_ standard was used to calibrate SDD and the relative tilt of the detector to the incident beam path. Two-dimensional diffraction patterns were integrated using FIT2D software ver. 18, developed at the European Synchrotron Radiation Facility (ESRF, Grenoble, France). The Match!3 software ver. 3.12 (Crystal Impact, Bonn, Germany) was used to compare the diffraction pattern of samples to reference patterns included in the Crystallography Open Database to identify the phases present before and after cultivation.

The complex data analysis was performed using Analysis ToolPak and Solver, statistical add-ins in Microsoft Office Excel (Redmond, WA, USA). Manganese extraction performances by the fungus were compared via *t*-test (two-sample assuming unequal variances) at a significance level α = 0.01.

## 3. Results

### 3.1. Fungal Resistance to Manganese(II) and Manganese Oxides

**Figure 1:**
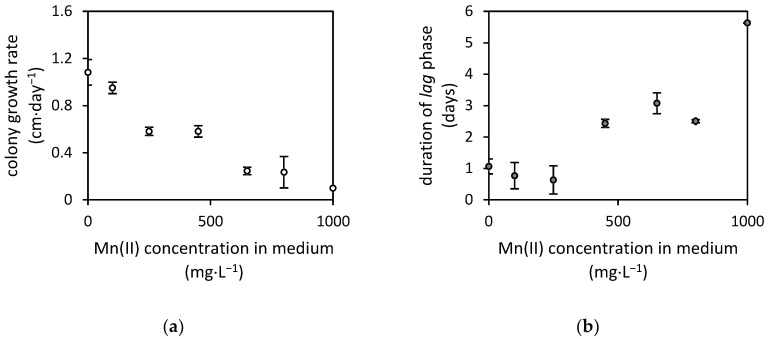
The effects of dissolved manganese(II) concentrations in agar media on (**a**) the colony growth rate of *Aspergillus niger* strain and (**b**) the duration of *lag* phase. The *lag* phase was estimated using modified Gompertz’s equation [15].

The *A. niger* strain was tested for its resistance to manganese by measuring the fungal colony growth on agar media. There, the fungus was exposed to up to 1000 mg·L^−1^ of manganese(II). Its sensitivity resulted in a decrease in colony growth rate (cm·day^−1^), and the *lag* phase was prolonged (Figure 1). The test concluded in the determination of 50% colony growth inhibitory concentration at 330 mg·L^−1^.

**Figure 2:**
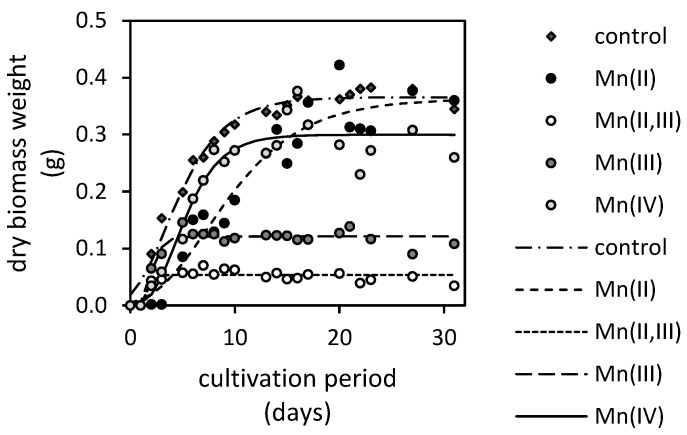
Changes in biomass weight of *Aspergillus niger* cultivated for 31 days in the culture medium supplemented with various manganese oxides (MnO, MnO·Mn_2_O_3_, Mn_2_O_3_ and MnO_2_; control is a manganese-free treatment). Experimental data were fitted using modified Gompertz’s equation [15].

The sensitivity of the *A. niger* strain to oxides and bioextracted manganese can be evaluated using the recorded changes of dry weights of fungal biomasses (Figure 2). The biomass harvested from the Mn(II)-oxide (MnO) treatment almost achieved the weight of the manganese-free control at the end of cultivation. It was followed by the biomass cultivated in the presence of the Mn(IV)-oxide (MnO_2_) and Mn(II)-oxide. The least amounts of biomasses were recorded in the presence of Mn(II,III)-oxide (MnO·Mn_2_O_3_). The length of *lag* phase of each treatment, which was calculated along the maximum growth rate using the modified Gompertz’s equation [15], indicated when a considerable cell division and, thus, the exponential growth phase occurred. It was the longest for the fungus cultivated in the presence of Mn(II)-oxide (2.3 days), followed by the Mn(IV)-oxide treatment (2.0 days). In the presence of Mn(II,III)-oxide and Mn(III)-oxide (Mn_2_O_3_), the exponential growth phase was achieved by the fungus significantly faster, after a day. The maximum growth rate was reduced only in the presence of Mn(II)-oxide (0.03 day^−1^). The rates estimated in the presence of all other oxides approximated the growth rate of fungus cultivated on manganese-free media (0.05 day^−1^).

### 3.2. Manganese Bioextraction

**Figure 3:**
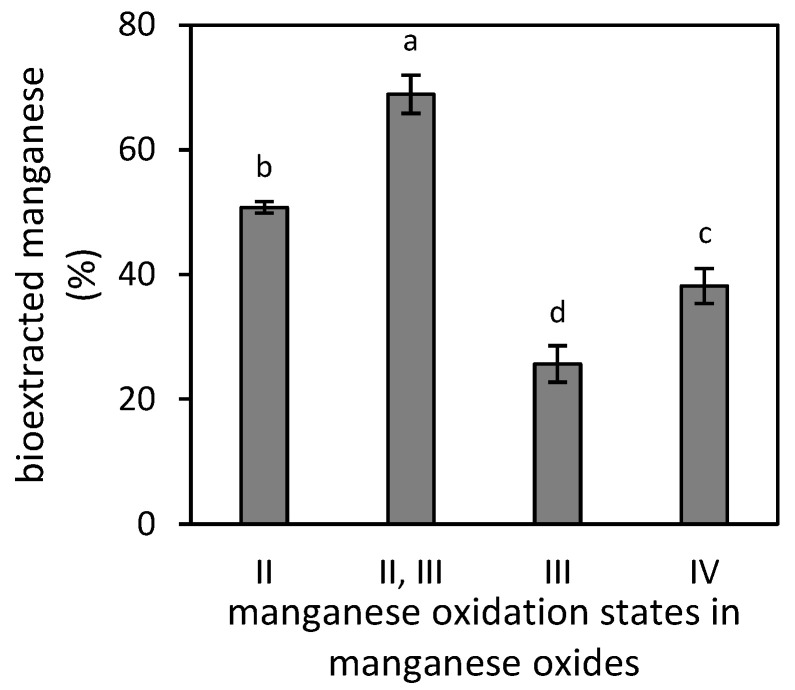
Bioextraction of manganese from its oxides (MnO, MnO·Mn_2_O_3_, Mn_2_O_3_ and MnO_2_) by microscopic filamentous fungus *Aspergillus niger* after a 21-day cultivation (initial weigh of each oxide was 0.25 g in 50 mL of culture medium; the extraction efficiencies were compared for all treatments via two-tail *t*-test at the 0.01 significance level).

The efficiencies of manganese bioextraction from various manganese oxides (MnO, MnO·Mn_2_O_3_, Mn_2_O_3_ and MnO_2_) by filamentous fungus *A. niger* were initially examined for the even 0.25 g weight of each oxide. The collected data, presented in Figure 3, revealed that the Mn(II,III)-oxide was the most susceptible to fungal deterioration, approximating 69% extraction efficiency. The Mn(III)-oxide was the most stable with less than 27% of manganese extracted after the 21-day cultivation.

The values of bioextraction efficiency along the calculated 50% inhibitory concentration were further used as reference values to calculate the initial amount of each oxide for further evaluation of bioextraction kinetics. In effect, the oxides’ weight that included the total amount of 330 mg manganese was increased by the non-extractable manganese estimated from Figure 3. The calculated amounts of oxides were subsequently supplemented in broth media and were exposed to growing fungus.

**Figure 4:**
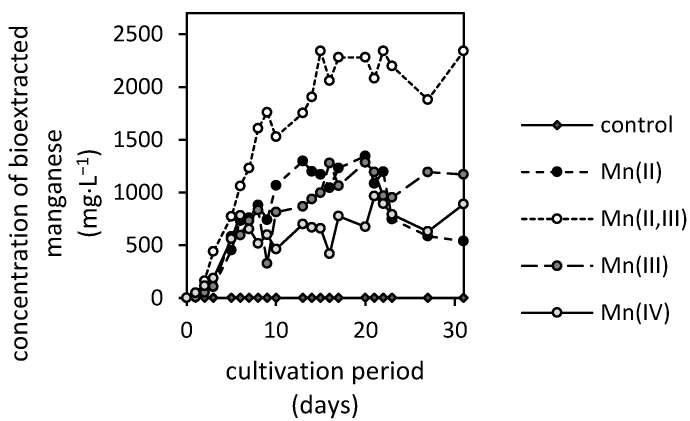
Concentrations of bioextracted manganese from its oxides (MnO, MnO·Mn_2_O_3_, Mn_2_O_3_ and MnO_2_) at different oxidation states during the static cultivation of microscopic filamentous fungus *Aspergillus niger*.

Figure 4 depicts that the filamentous fungus *A. niger* was capable of mobilizing manganese from all oxides. Since the extraction efficiencies in non-inoculated control during a 31-day experiment were negligible, it was concluded that the manganese oxides’ deterioration and subsequent stabilization of extracted manganese in solution resulted solely from fungal activity.

The extraction kinetics revealed various distinctive phases that are similar to the characteristics of microbial growth. The slow initial phase is followed by the steep increase in manganese extraction that is subsequently diminished after the 10th cultivation day (Figure 4). These are the general features of Mn(II,III), Mn(III) and Mn(IV)-oxides’ biodeterioration. There, the Mn(II,III)-oxide was the most susceptible to fungal activity, reaching the 26% extraction efficiency (2340 mg·L^−1^). It was followed by Mn(II), Mn(III) and Mn(IV)-oxides, which did not display a statistically significant difference in maximum amounts of extracted manganese due to high variabilities in observed values. However, the average maximums of manganese in culture medium were 1210, 1140 and 700 mg·L^−1^ for Mn(II), Mn(III) and Mn(IV)-oxides, respectively.

The deterioration of Mn(II)-oxide featured the specific phase where the manganese concentrations seemingly decreased in the medium. This was characteristic in the late growth phase of the fungus and coincided well with other measured growth parameters, including biomass weight, pH and depletion of extracellular low-molecular-weight organic acids.

### 3.3. Changes in pH and Concentrations of Low-Molecular-Weight Organic Acids in the Presence of Manganese Oxides

During the 31-day cultivation, fungus *A. niger* was capable of producing the extraction-inducing extracellular metabolites to various extents. These, in the course of time, have changed the qualitative and quantitative parameters of culture media, ultimately altering the extraction performance of fungus towards each manganese oxide. Capillary isotachophoresis revealed that, during cultivation, the low-molecular-weight organic acids of oxalate and gluconate were primarily produced by the fungus.

**Figure 5:**
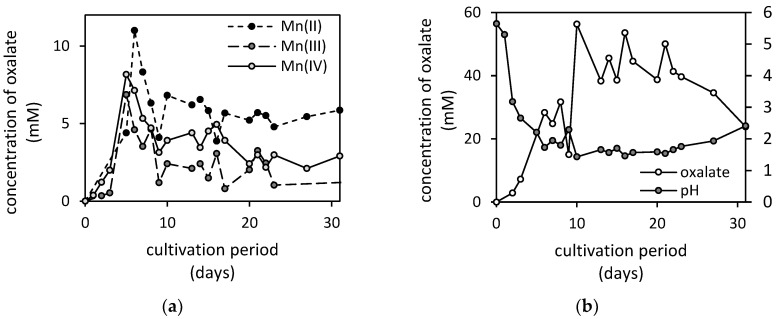
Changes of oxalate concentrations in culture media supplemented with (**a**) Mn(II); Mn(III) and Mn(IV)-oxides, and (**b**) changes of pH and oxalate concentrations in manganese-free treatment (control) during a 31-day cultivation of fungus *Aspergillus niger*.

The concentration of oxalate accumulated in the culture medium, after being exposed to Mn(II)-oxide, reached its maximum of 11 mM on the 6th day of cultivation. Thereafter, the oxalate concentration in the culture medium steeply decreased and remained relatively stable for the rest of the cultivation (Figure 5). A similar pattern, with slightly lower extracellular oxalate concentrations, was also observed after exposure to Mn(III) and Mn(IV)-oxides (Figure 5). No oxalate was detected in the presence of Mn(II,III)-oxide. It is also worth mentioning that the accumulation of oxalate in the culture medium was significantly higher in a manganese-free treatment, and it reached the maximum concentration of 56 mM.

**Figure 6:**
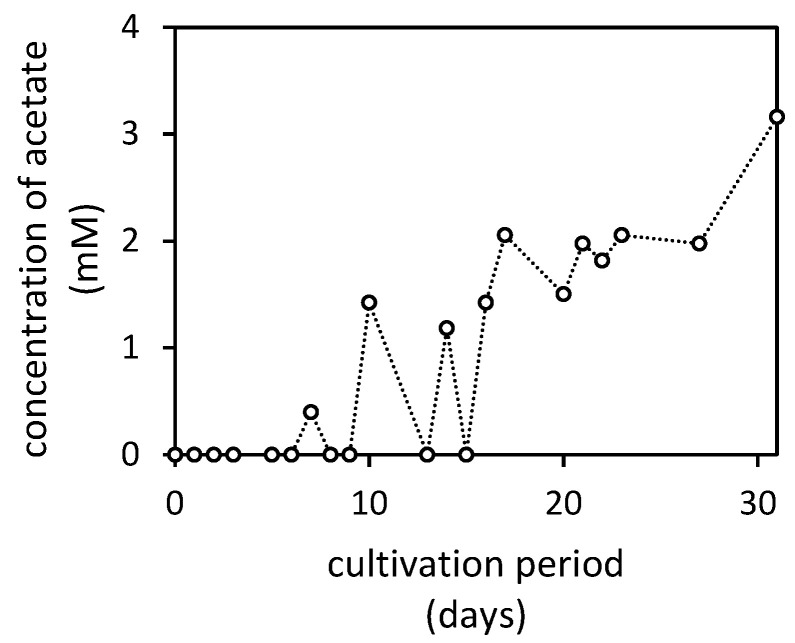
Changes in acetate concentration in culture medium supplemented with Mn(IV)-oxide (MnO_2_) incubated in the presence of *Aspergillus niger* for 31 days. The concentration of acetate in other treatments was below the detection limit (0.004 mM) of applied analytical procedure.

The presence of acetate was noted only in culture media supplemented with the Mn(IV)-oxide (Figure 6). In all other treatments, the fungus did not produce any acetate, or it was consumed/degraded during the cultivation. Alternatively, its concentration was below the detection limit of the applied analytical procedure.

**Figure 7:**
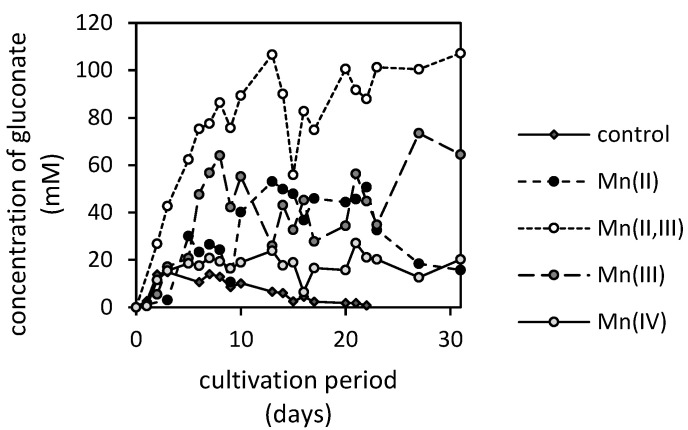
Changes in gluconate concentration in culture medium supplemented with various manganese oxides (MnO, MnO·Mn_2_O_3_, Mn_2_O_3_ and MnO_2_) incubated in the presence of *Aspergillus niger* for 31 days (control is a manganese-free treatment).

Notable changes in the concentration of gluconate have been observed after exposure to various manganese oxides (Figure 7). The fungus was capable of accumulating and metabolizing gluconate to various extents during cultivation. The apparent gluconate accumulation was the lowest in the case of the manganese-free control, while the highest concentration in the culture medium was observed in the Mn(II,III)-oxide treatment (over a 100 mM concentration). Since gluconate anions are derived from glucose extracellularly and are subsequently uptaken, it is likely that the synthesized gluconate in the case of the control experiment was completely metabolized in later growth phases and, thus, no gluconate was detected in the culture medium after the 22nd day of cultivation.

Significant depletion of gluconate after three weeks was also noted in the case of Mn(II)-oxide, where the 50 mM of gluconate decreased within a few days below 20 mM concentration. The Mn(III)-oxide led to a higher accumulation of gluconate in the early and late stages of fungal growth, approximating 64 and 73 mM concentrations, respectively. The exposure of fungus to Mn(IV)-oxide activated extensive gluconate utilization, thus, its concentration in the medium was low and relatively stable with values reaching up to 18 mM (Figure 7).

**Figure 8:**
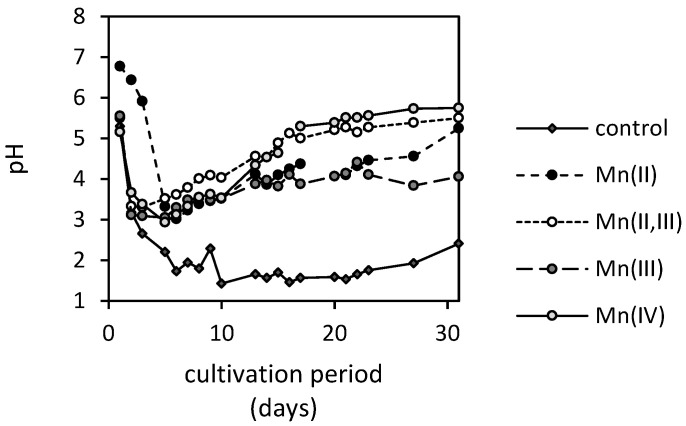
Changes in pH of culture medium supplemented with various manganese oxides (MnO, MnO·Mn_2_O_3_, Mn_2_O_3_ and MnO_2_) incubated in the presence of *Aspergillus niger* for 31 days (control is a manganese-free treatment).

The culture media pH in the presence of manganese oxides fluctuated between the values of six and three (Figure 8). Initial acidification within the first five days provided ideal conditions for (reductive) dissolution of manganese oxides (Figure 4). Thereafter, the pH gradually increased with Mn(II,III) and Mn(IV)-oxides, reaching a pH of five on the 16th day of cultivation. The pH values of culture media treated with Mn(II) and Mn(III)-oxides ascended to the value of five significantly slower. The manganese-free treatment indicated inhibitory effects of manganese on proton exudation since it decreased its pH below the value of two and remained so almost until the end of cultivation. There, it increased to the value of 2.4.

### 3.4. Formation of Biogenic Mineral

**Figure 9:**
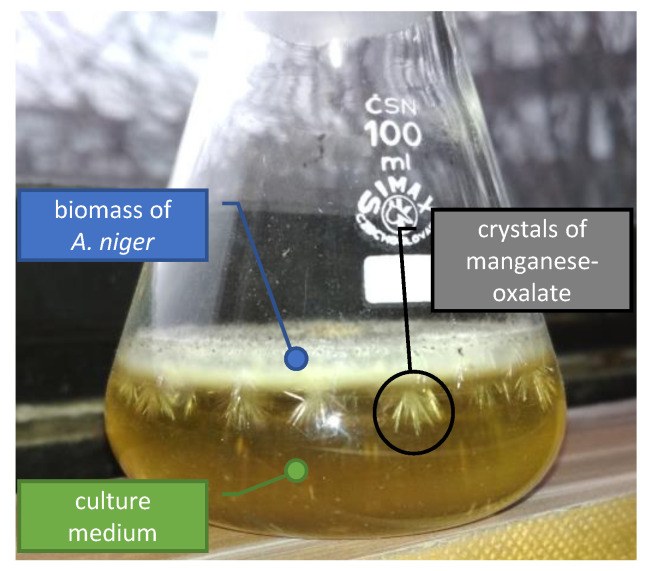
The biogenic manganese oxalate crystals associated with the fungal biomass of filamentous fungus *Aspergillus niger* cultivated in the presence of Mn(IV)-oxide.

**Figure 10:**
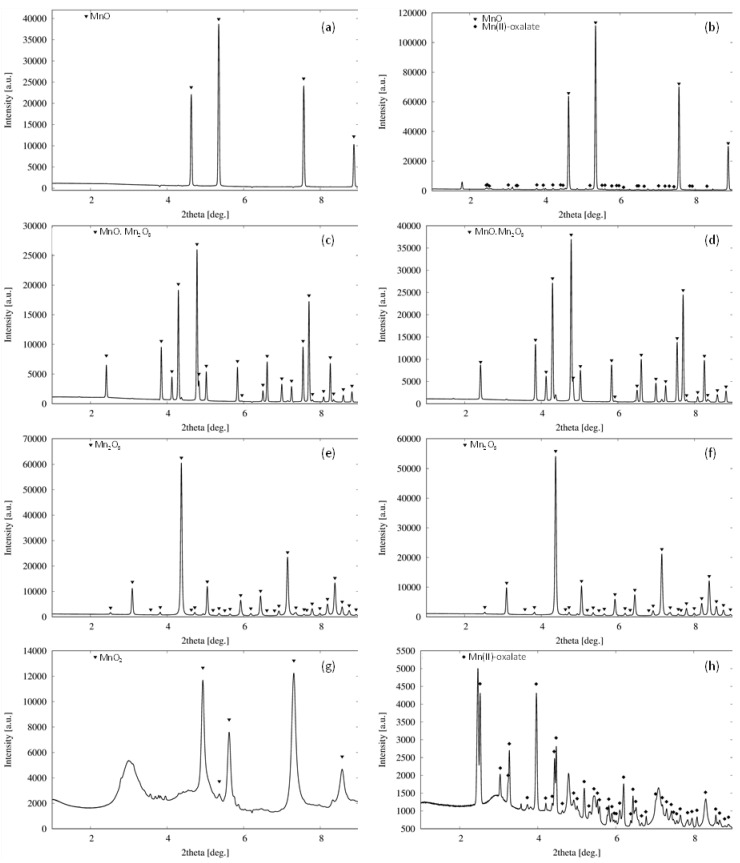
High energy (synchrotron) X-ray diffraction of Mn(II), Mn(II,III), Mn(III) and Mn(IV)-oxides (**a**,**c**,**e**,**g**) at the initiation of a 31-day incubation in the presence of filamentous fungus *Aspergillus niger*, and (**b**,**d**,**f**,**h**) at the end of cultivation in respective order.

During the cultivation, a large crystal formation in the biomass of microscopic filamentous fungus was observed (Figure 9). It was most apparent in media supplemented with Mn(IV) and Mn(II)-oxides, while the exposure of fungus to Mn(III) and Mn(II,III)-oxides did not result in the detectable formation of new mineral phases. While the initial crystallinity of Mn(II), Mn(II,III) and Mn(III)-oxides was relatively high, the sample of Mn(IV)-oxide was less ordered, and it included amorphous phases and a small fraction of manganese-oxides at different oxidation states as well (Figure 10).

The formation of crystals was noticeable after the seventh cultivation day (Figure 9), and it correlated well with the decrease and fluctuations of oxalates’ concentrations in the culture media (Figure 5). There, the extracted manganese and accumulated oxalate concentrations reached the required amounts for precipitation of new biomineral phases. The newly formed crystals were analyzed by synchrotron X-ray diffraction analysis at the end of cultivation, and they were identified as manganese oxalates (Figure 10). Thus, the formation of manganese oxalates during the cultivation experiments was confirmed in systems supplemented with Mn(IV) and Mn(II)-oxides.

## 4. Discussion

Our results (Figure 4) highlighted that the fungus *A. niger* was capable of rapid disintegration of naturally occurring manganese oxides. Since the abiotically extracted manganese did not reach the culture medium concentration of 0.15 mg·L^−1^ in the non-inoculated control, we have concluded that the oxides are highly stable in near-neutral abiotic systems, and the fungal strain’s presence is essential for the extensive mobilization of manganese. In the case of Mn(II,III)-oxide, the fungus was capable of dissolving 26% of manganese within the 31-day cultivation. This resulted in its significant accumulation in the culture medium, reaching a concentration of 2340 mg·L^−1^.

Since the fungus has shown a capacity to solubilize metal oxides that are natural components of geochemical barriers [17,18], its activity in the environment has various undesired impacts. The severity of this process is underlined by the fact that manganese oxides serve as a natural scavenger of various potentially toxic compounds, as well as trace metals in soils and sediments [19]. Thus, our research, which confirmed the fungal ability to dissolve all of the naturally occurring manganese oxides, emphasizes the potential negative impacts of fungal activity in contaminated soils and sediments regarding the mobility of hazardous elements [20].

On the other hand, the solubilization of manganese oxides by filamentous fungi shows immense potential for the recovery of manganese from discarded low-grade manganiferous ores since it is considered a better green alternative to pyrometallurgical and hydrometallurgical techniques [21]. Archarya et al. [22] studied the bioleaching of manganese ore (2% *w*/*v*) using culture solutions of *Penicillium citrinum* strain. The secreted fungal metabolites were capable of removing 19.6% of manganese within a period of 30 days. This is similar to the extraction performance of *A. niger* in our study. However, Mehta et al. [23] reported that the *A. niger* strain was capable of extracting up to 91% of manganese from oceanic polymetallic nodules (5% *w*/*v*). The reported bioleaching efficiency highlighted the potential of *A. niger* for manganese bioextraction from environmental samples. This process was recently optimized by Keshavarz et al. [24] for the bioleaching of low-grade pyrolusite (pulp density of 1 g·L^−1^) with the extraction maximum of 78.8% achieved through a two-step method using the medium collected after 21 days of dynamic cultivation (120 rpm) of fungus in the glucose medium. The strains of *Aspergillus* section *Nigri* also proved to be an efficient extractor of manganese from Mn-Zn batteries [25]. Still, none of these studies distinguished the effect of the manganese oxidation state on bioextraction performance, which is reported in our study. However, in contrast to all other referred studies, our experimental leaching of manganese oxides was performed under static conditions as a one-step method. It is very likely that the static cultivation hindered the mobility of nutrients and the aeration of media, thus, the fungal activity could be altered unfavorably in comparison to dynamic cultivation. Furthermore, the two-step bioextraction method is more advantageous, since the direct exposure to extracted manganese through the one-step method adversely affected the fungal growth (Figure 2). Still, in the case that the fungal growth was not affected, the extraction performance of the two-step method under both dynamic and static conditions tends to be similar [14]. Nevertheless, the indirect exposure of manganese phases to fungal metabolites via the two-step method is most likely the main reason why the experimental methods of other authors outperformed our extraction efficiency.

One of the principal mechanisms of manganese mobilization seems to be the synthesis of extracellular low-molecular-weight organic acids with exceptional chelating and acidic properties [26]. However, fungi usually excrete these in the dissociated form since the intracellular pH is at neutral levels [27]. Therefore, the extremely low pH of the extracellular environment (below 3.5, Figure 8) should coincide with the intensive fungal extrusion of H^+^ to maintain the neutral intracellular pH [28].

Based on our previous experiences, we assumed that, besides proton extrusion, the oxalate would be the key metabolite in the disintegration of manganese oxide minerals [17,29]. Therefore, the outcome that the Mn(II,III)-oxide was the most intensively biodeteriorated, despite no oxalate being produced by the fungus during incubation, was unexpected. Since oxalate accumulation in the medium was one magnitude higher in the manganese oxide-free treatment compared to other systems (Figure 5b), and extreme acidification of media should reportedly not contribute to the enhancement of oxalate synthesis [30], we concluded that the presence of high manganese concentration was the primary factor that inhibited oxalate efflux from the fungal cell. Thus, the absence of oxalate in the Mn(II,III)-oxide treatment was likely caused by the excessive release of manganese via acidolysis.

This should allow us to conclude that it is very likely that the acidification of culture media played a key role in manganese oxides’ dissolution. However, our observations also suggest an alternative or co-occurring pathway of manganese oxides’ deterioration—a reductive dissolution. Xyla et al. [31] noted that, during the extraction of manganese from Mn(IV)-oxide (*β*-MnO_2_), the oxalate and oxide are consumed in a ratio of 1:1. The protons are also consumed; thus, the manganese(II) release from Mn(IV)-oxide follows the Equation (1):

MnO_2_ (s) + C_2_O_4_^2−^ + 4H^+^ ↔ Mn^2+^ + 2CO_2_ + 2H_2_O. (1)

Therefore, the reductive dissolution of Mn(IV)-oxide is facilitated under acidic conditions and by the occurrence of oxalate. Oxalate forms a precursor surface complex that results in an electron transfer and a release of the reduced manganese(II) ion to the solution [31]. This explains the high extraction efficiency at higher pHs and at apparent oxalate absence in a medium supplemented with Mn(II,III)-oxide in the presence of a fungus.

There are also reports suggesting the direct involvement of enzymatic activity in redox transformation of manganese by filamentous ascomycetes. Miyata et al. [32] reported that the *Acremonium* KR21-2 strain was capable of oxidizing dissolved Mn(II) via the activity of a laccase- or multicopper oxidase-like protein that is distinct from the basidiomycete laccases [33]. Recently, Zeiner et al. [34] revealed the presence of both Cu-based and FAD-based Mn(II) oxidation mechanisms in various ascomycete species and suggested candidates for oxidases, including tyrosinase, glyoxal oxidase, bilirubin oxidase, and glucose-methanol-choline oxidoreductase. The oxidative transformation of Mn(II) should lead to the deposition of (biogenic) Mn(III/IV)-oxides [35]. However, besides biogenic oxalates, no other new mineral phases have been detected in the presence of Mn(II), Mn(II,III) and Mn(III)-oxides (Figure 10). While enzymatic reductive dissolution of manganese oxides is characteristic primarily for bacterial species [36], filamentous fungi most likely mediated the reduction of manganese non-enzymatically [37], for example, via oxalate or citrate [31,38,39].

Exposure of fungus to excessive amounts of manganese also alters the apparent gluconate accumulation in the medium, however, inversely to oxalate (Figure 7). It is well known that gluconate is derived from glucose due to glucose oxidase activity [40], and it can serve as an easily utilizable carbon source for *A. niger* [41]. The gluconate synthesis at a pH lower than 3.5 is significantly inhibited, since the enzyme’s activity is dependent on external pH [42] with an optimum at pH 6 [43]. This is in good agreement with our experimental findings (Figure 7), where the accumulation of gluconate was insignificant in the case of manganese-free treatment with the lowest pH level. On the other side, the highest accumulation in the medium was observed in the cultivating system treated with Mn(II,III)-oxide, where the pH was almost continuously above the value of 3.5 (Figure 8). However, this can also indicate the negative impact of manganese on the metabolism of external carbon sources. It can be concluded that the rate of gluconate production is similar to that of gluconate uptake by biomass in the manganese-free treatment. Since the latter is not strictly dependent on the control mechanism as carbon catabolite repression [44], it is very likely that the significantly higher accumulation in culture media supplemented with Mn-oxides resulted from the unknown mechanism restricting gluconate uptake by fungus in manganese excess.

During the cultivation, the growth of biogenic crystals associated with the biomass of filamentous fungus was noted. The formation of crystals was observable from the seventh cultivation day and is associated with apparent fluctuation in free oxalate concentrations in the medium (Figure 5). There, the dissolved manganese and oxalate concentrations reached the required level for nucleation and subsequent biogenic mineral growth into a detectable manganese oxalate crystal associated with the biomass (Figure 9). Similarly, Ferrier et al. [45] identified the presence of lindbergite (manganese oxalate dihydrate) in the solid phases, forming sheaths around the hyphae of *A. niger* cultivated in the presence of manganese-rich deep-sea nodules. It was most apparent in culture media supplemented with Mn(IV)-oxide (Figure 9), followed by the Mn(II)-oxide, while for all other cases it was unnoticeable. The absence of crystal formation in Mn(II,III) and Mn(III)-oxide treatments can be explained by the extensive consumption of oxalate due to reductive dissolution.

Furthermore, the ability to form mineral oxalates provides the fungus with an ecological advantage in contaminated soils and sediments. There, the oxalates can serve as a detoxification agent due to their sorptive properties and ability to coprecipitate some of the heavy metals, including manganese. It allows the fungus to outcompete other microorganisms for this ecological niche [46]. This is due to formation of highly stabile biogenic oxalates that decrease the exposure of microorganisms to potentially toxic manganese [47] or other metals [48]. This is also highlighted by our results, since the formation of manganese oxalates were shown to alleviate the stress related to the bioextraction of manganese. This has manifested in both Mn(II) and Mn(IV)-oxides treatments (Figure 2). There, the growth of the fungus was initially inhibited (in comparison to the manganese-free control). However, after the formation of manganese oxalates, the growth was restored and the final dry biomasses’ weights were comparable to the control. Still, since the detectable amounts of acetate were recorded in media treated with Mn(IV)-oxide, where the presence of manganese oxalate was most noticeable, we hypothesized that the formation of biogenic phases associated with fungal biomass (Figure 9) might also have a negative impact on fungal metabolism or cell integrity. Ruijter et al. [30] reported that the acetate should be consumed sufficiently fast to prevent its accumulation. This suggests that the overproduction of acetate along with the oxalate via oxalacetate cleavage took place, and while oxalate was being incorporated into growing crystals, the acetate needed to be extruded to lighten the burden on the cell. Alternatively, the growing crystal may have physically deformed the cell membrane, resulting in leakage of acetate (alongside the other metabolites) into the culture medium.

The noted manganese recrystallization is also a potential problem for the commercial application of low-grade ores’ bioleaching by filamentous fungi since it can hinder the bioextraction performance due to undesirable manganese immobilization into a biogenic thermodynamically stable mineral (e.g., lindbergite). Still, Li et al. [49] reported that the manganese biomineralization can be also a useful strategy for the synthesis of novel electrode materials.

## 5. Conclusions

Our experimental observations confirmed that filamentous fungus *A. niger* was capable of dissolving all naturally occurring manganese oxides (MnO, MnO·Mn_2_O_3_, Mn_2_O_3_, MnO_2_) and that it can act as a geoactive agent regarding manganese transformation and mobilization in the environment. We have successfully simulated the process of fungal deterioration of Mn(II), Mn(II,III), Mn(III) and Mn(IV)-oxide minerals via mechanisms of acidolysis, complexolysis and reductive dissolution. Through the metabolic activity, the fungus *A. niger* showed the ability to condition the cultivation system to the extent that it resulted in the transformation of manganese into mycogenic mineral. This should inherently affect the stability of manganese oxides present in geochemical barriers, and the migration and distribution of other elements associated with their surfaces. Therefore, further research on the sorption properties of the natural manganese oxides and their transformants is required to better understand the environmental impact of fungal bioleaching. Thus, our results highlight the importance of fungi in biogeochemical cycling and the environmental behavior of manganese, and provide insight into the actual process of manganese oxides’ biodeterioration. 

## Data Availability

Data is contained within the article.
